# Changes in lumbar kinematics and trunk muscle electromyographic activity during baseball batting under psychological pressure

**DOI:** 10.1080/23335432.2020.1811765

**Published:** 2020-08-20

**Authors:** Tomoki Oshikawa, Yasuhiro Morimoto, Gen Adachi, Hiroshi Akuzawa, Koji Kaneoka

**Affiliations:** a Graduate School of Sport Sciences, Waseda University, Saitama, Japan; b Faculty of Sport Sciences, Waseda University, Saitama, Japan

**Keywords:** Baseball batting kinematics, lumbar spine, electromyography, trunk muscle, different psychological pressure conditions

## Abstract

Psychological pressure during sports competition disturbs the ideal physical movement and causes injury. Baseball batting frequently causes trunk injuries. This study aimed to examine the influence of psychological pressure on the lumbar kinematics and trunk muscle activity during the baseball batting. Fourteen collegiate baseball players participated in this study. The participants performed bat swings under three different psychological conditions (non-pressure, pressure, and emphasized pressure). The lumbar kinematics and trunk muscle activity were measured during each bat swing. One- and two-way analyses of variance were performed to compare the lumbar kinematics and trunk muscle activity among different psychological pressure conditions. The lumbar flexion angle throughout the bat swing in the swing phase, from the moment of ground contact of the lead foot to the moment of ball contact, was significantly larger under the pressure and emphasized pressure conditions than under the non-pressure condition (P<0.05). The bilateral lumbar erector spinae (LES) activities in the swing and follow-through phases were significantly higher under the emphasized pressure condition than under the non-pressure condition (P<0.05). These results indicate that the baseball batting under psychological pressure influenced the lumbar kinematics and bilateral LES activities and may be related to the development of low back pain.

## Introduction

Psychological pressure during sports competition disturbs the ideal physical movement and causes injury. Previous reports showed that the incidence of muscle strain in sports is higher during competition than during practice (Dalton et al. 2015; Johnson and Comstock 2017; Eckard et al. 2017a, 2017b; Côrte et al. 2019). These reports may be attributable to the need for higher-intensity physical movements and stronger muscle contraction to achieve larger or faster movements for better results during competition (Dalton et al. 2015; Eckard et al. 2017a). In baseball batting, a faster bat tip velocity is important for hitting a high-velocity ball, which can travel a great distance (Sawicki and Hubbard 2003). Therefore, baseball players try to achieve a faster bat tip velocity by rotating the trunk rapidly with their full effort in important situations in the game. Although rotating the trunk rapidly with full effort is expected to increase bat tip velocity, a high-intensity batting action may cause an injury to the trunk.

The injuries frequently caused by the batting action are low back pain (LBP) (Tasaka et al. 2016) and abdominal muscle strain (Conte et al. 2012). A previous injury report on Major League Baseball players in the disabled list stated that the commonly injured body areas were the shoulder, elbow, and trunk, including LBP and abdominal muscle strain, in descending order of frequency (Conte et al. 2016). We speculate that the rapid trunk rotation in the batting action leads to mechanical stress on the lumbar facet joints (Sairyo et al. 2005), which are not suitable for rotation owing to their shape (Kapandji 2008). Strong tension in the external oblique (EO) and internal oblique (IO) muscles due to instantaneous contraction also occurs because pelvic rotation precedes thoracic rotation in the batting action (Welch et al. 1995; Escamilla et al. 2009). Excessive contraction may cause muscle strain. When baseball players increase the bat tip velocity, the pelvic rotation further precedes the thoracic rotation. The separate rotation between the pelvis and the thorax is believed to exert stress on the lumbar spine and abdominal muscles (Fleisig et al. 2013). Therefore, the stress on the lumbar facet joints, and EO and IO muscles is expected to increase under psychological pressure conditions that produce increased bat tip velocity.

Previous studies that investigated changes in physical movement under psychological pressure reported an increase in angular velocity in shoulder adduction on the back side (Tanaka and Sekiya 2010) and electromyographic (EMG) activity of the extensor carpi radialis on the lead side (Cooke et al. 2010) during golf putting. In addition, Yoshie et al. (2009) reported an increase in biceps brachii and upper trapezius activity under psychological pressure during piano performance, although different from the movements in sports. However, no studies have investigated whether psychological pressure to increase bat tip velocity changes physical movement during the baseball batting action. Investigating the influence of psychological pressure on lumbar kinematics and the EMG activity of the trunk muscles can help clarify the mechanism of the injuries caused by the batting action.

This study aimed to examine the influence of psychological pressure on the lumbar kinematics and EMG activity of the trunk muscles during the batting action. We compared the lumbar kinematics and EMG activity of the trunk muscles during the batting action among three different psychological pressure conditions. Our hypothesis was that the instantaneous peak angle of lumbar rotation, peak angular velocity of lumbar rotation toward both the lead and back sides, and EMG activities of the EO and IO muscles would be higher under psychological pressure conditions than under non-pressure condition.

## Materials and methods

### Participants

Fourteen male collegiate baseball players (age: 21 ± 1 years, height: 173.4 ± 3.6 cm, weight: 74.6 ± 5.3 kg, baseball experience: 13 ± 1 years) participated in this study. Their baseball training was 4 hours per day, 6 days per week on an elite collegiate baseball team. The participants did not have injuries that limited their practice within the last 2 years. To create the psychological pressure conditions that increase bat tip velocity, we informed the participants before the experiment that we would reward them 20 USD for participation in the experiment. The reward of 20 USD was set in accordance with previous studies (Tanaka and Sekiya 2010 [2000 JPY]; Cooke et al. 2010 [mean reward £15]) that investigated changes in kinematics and EMG activity under pressure conditions. A power analysis was performed for two-way analysis of variance (ANOVA; phases × conditions) using G*Power 3.1 (Heinrich-Heine Universität, Germany). The number of participants was estimated to be 10, with an alpha value of 0.05, power of 0.95, and partial *η^2^* for effect size of 0.14. This study was approved by our institutional ethics committee (approval No. 2016–306). The experimental protocol was explained to all the participants, who agreed to participate and provided informed consent prior to the experiment.

### Electromyography

The EMGs of the rectus abdominis (RA), EO, IO, lumbar erector spinae (LES), and lumbar erector multifidus (LMF) muscles were recorded using surface electrodes applied bilaterally. Before the attachment of the electrodes, the skin was abraded with an abrasive and alcohol to reduce skin impedance to <2 kΩ. Surface electrodes of 8 mm in diameter (BlueSensor N-00-S, METS Co., Japan) were attached to each muscle belly, parallel to the muscle fiber. The electrode was placed 3 cm lateral to the umbilicus for the RA (Stevens et al. 2007; Okubo et al. 2010b), 15 cm lateral to the umbilicus for the EO (Stevens et al. 2007; Okubo et al. 2010b), 1 cm medial and downward to the anterior superior iliac spine for the IO (Ng et al. 1998; Preuss et al. 2005), 3 cm lateral to the L3 spinous process for the LES (Preuss et al. 2005; Okubo et al. 2010b); and 2 cm lateral to the L5 spinous process for the LMF (Kavcic et al. 2004; Okubo et al. 2010a). The distance between the electrodes was 20 mm. A wireless EMG telemeter system (BioLog DL-5000, S&ME Co., Japan) with a sampling rate of 1000 Hz was used to measure the EMG activity.

### Experimental protocol

Following the optional 10-minute warm-up with many bat swings by each participant, all surface electrodes were attached, and the maximum voluntary isometric contractions (MVICs) for each trunk muscle were recorded to normalize the EMG data. For the RA, the participants performed trunk flexion in the crook-lying position with manual resistance applied to the anterior shoulder aspect in the trunk extension direction. For the EO and IO, the participants performed trunk flexion and right or left rotation in the crook-lying position, with hands in front of the chest. Manual resistance was applied at the shoulder in trunk extension and left or right rotation. For the LES and LMF, the subjects performed trunk extension in the prone position with manual resistance applied to the posterior shoulder aspect in the trunk flexion direction. The MVIC trials of each muscle were performed for 5 seconds. The resting time between each MVIC trial was more than 1 minute.

**Figure 1.**
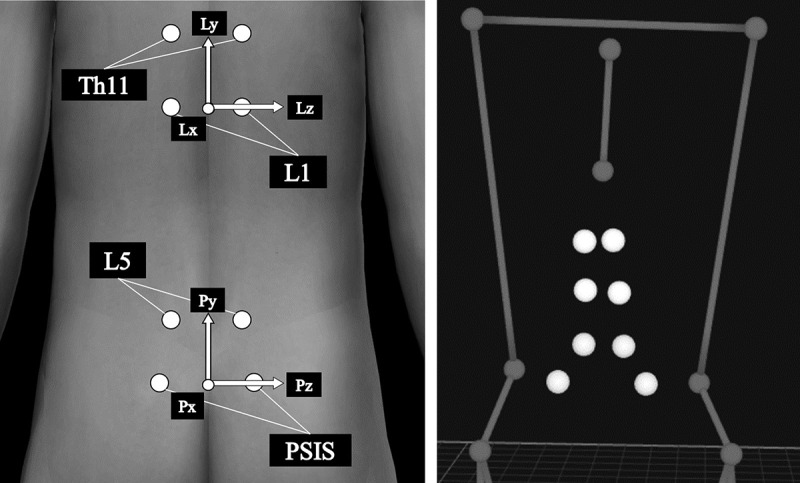
Markers for the lumbar kinematics measurement (Left: body surface model, Right: three-dimensional motion analysis model). Pelvic and lumbar coordinate systems are configured from these markers. The lumbar kinematics is defined as (+) lumbar extension/(−) flexion on the *z*-axis, (+) lumbar rotation toward the lead side/(−) back side on the *y*-axis, and (+) lumbar lateral flexion toward the back side/(−) lead side on the *x*-axis

After the MVIC tests, 16-mm reflective markers (Qualisys AB, Sweden) were attached to the body landmarks and both ends of the bat. To measure lumbar kinematics during the bat swing, markers were also attached to the posterior superior iliac spine (PSIS) and 2 cm lateral to the spinous processes of Th11, L1, and L5 (Oshikawa et al. 2020) (Figure 1).

**Figure 2.**
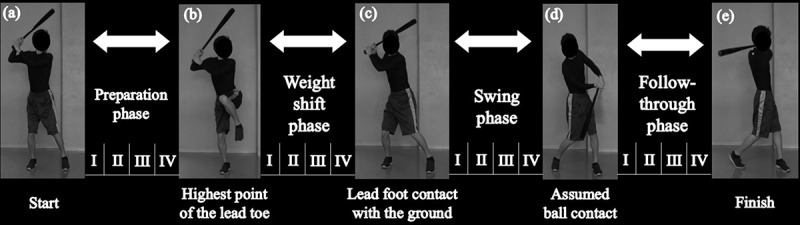
The bat swing is divided into four phases based on five key events. To compare the lumbar angle throughout the bat swing, the angular data are converted into 100 data points in each phase, evenly divided into 25 data points, and four subphases (I–IV)

All the trials were recorded using eight three-dimensional (3-D) motion capture cameras (OQUS, Qualisys AB, Sweden) at a sampling rate of 200 Hz (Oshikawa et al. 2020). First, to measure the static lumbar alignment during the anatomical standing posture, the participants were instructed to stand with palms forward and feet apart at shoulder width for 10 seconds. Second, we asked them to maintain their batting posture at the moment of ball contact for 10 seconds (assumed ball contact, Figure 2(d)). This trial measured the angle formed by the lines of both the anterior superior iliac spine (ASIS) markers and ends of the bat (ASISs-bat angle) and was used to define the swing phases. For the participants to perform the bat swing trials under different psychological pressure conditions, we created three different psychological circumstances as follows: (1) Non-pressure condition: We instructed the participants to ‘perform five swings similar to their usual practice.’ (2) Pressure condition: We told the participants that we would compare the mean bat tip velocity of the next five swings with that under the non-pressure condition, and increase or decrease their reward by ±1 USD per bat tip velocity ±1 km/h. (3) Emphasized pressure condition: After showing the participants a false result in which the mean bat tip velocity of five swings under the pressure condition exceeded that under the non-pressure condition by 3 km/h, we informed the participants that if the mean bat tip velocity of the next five swings exceeded that under the pressure condition, we would increase their reward further. State-trait anxiety inventory (STAI) Y-1 was used to measure the participants’ psychological state anxiety after the instruction of each condition was given. The participants performed practice swings in all the conditions without batting a ball, using a designated bat (length, 84 cm, weight, 890 g, maximum diameter, 64 mm, Mizuno Co., Ltd., Japan). In addition to batting practice, baseball players spend considerable time taking practice swings without a ball (Nara et al. 2009). When a player hits a ball, the technical elements of reacting to the timing of the ball cause a change in the batting movement; thus, the repeatability of the kinematic data is difficult to ensure. Therefore, the participants performed practice swings in their own timing, and we measured the natural lumbar kinematics during practice swings without the influence of hitting a ball (Oshikawa et al. 2020). Furthermore, because each participant’s own bat has a different length (78–86 cm) and weight (780–950 g), the same bat was used to minimize the influence of the characteristics of the bat on lumbar kinematics. After the trials, a psychological debriefing revealed the false information provided on the bat tip velocity necessary for the experiment, and a reward of 30 USD was paid to all the participants.

### Dividing the bat swing into phases

Swing motion was divided into four phases (preparation, weight shift, swing, and follow-through) based on five key events according to previous studies (Washington and Oliver 2018; Oshikawa et al. 2020) (Figure 2). The preparation phase was defined as the period from the moment the lead foot was lifted off the ground to the moment the marker on the lead toe reached its highest point. The weight shift phase was defined as the period from the end of the preparation phase to the moment the lead foot contacted the ground again. The swing phase was defined as the period from the end of the weight shift phase to the moment of assumed ball contact. The moment of assumed ball contact was defined as when the ASIS-bat angles in the assumed ball contact and bat swing trials were closest in value. The follow-through phase was defined as the period from the assumed ball contact to the end of the swing.

### Motion analysis

Lumbar kinematics was calculated using the Cardan angles (Oshikawa et al. 2020) from the lumbar coordinate system orientation relative to the pelvic coordinate system. The pelvic coordinate system was configured from the markers on the PSIS and L5. The pelvic transverse axis (Pz) was directed from the midpoint of both PSISs to the right PSIS. The temporal vertical axis (Tem-Py) was directed from the midpoint of both PSISs to the midpoint of both L5. The sagittal axis (Px) was defined as the cross-product of Tem-Py and Pz. The vertical axis (Py) was defined as the cross-product of Pz and Px. Similar to the calculation method for the pelvic coordinate system, the lumbar coordinate system (Lx, Ly, and Lz) was configured from the markers on L1 and Th11 (Figure 1). Lumbar kinematics was defined as (+) lumbar extension/(−) flexion on the *z*-axis, (+) lumbar rotation toward the lead side/(−) the back side on the *y*-axis, and (+) lumbar lateral flexion toward the back side/(−) the lead side on the *x*-axis (Figure 1, Table 1).

**Table 1.** Calculating method for the Cardan angles

*I*′	*J*′	*K*′
cosθcosφ	−sinθcosψ + cosθsinφsinψ	sinθsinψ + cosθsinφcosψ
sinθcosφ	cosθcosψ + sinθsinφsinψ	−cosθsinψ + sinθsinφcosψ
−sinφ	cosφsinψ	cosφcosψ

θ: (+) lumbar extension/(−) flexion, φ: (+) lumbar rotation toward lead side/(−) back side, ψ: (+) lumbar lateral flexion toward back side/(−) lead side

*I′, J*′, and *K*′ are the unit vectors after calculating the product of the matrix around each axis.

The lumbar angles during bat swing were normalized by calculating the differences between the lumbar angles during the anatomical standing posture and each angular data point during the bat swing. From these angular data and bat tip marker, the following outcomes were analyzed on the basis of previous studies (McGregor et al. 1997; Dowling and Fleisig 2016): the instantaneous peak angle and ROM of the lumbar spine, peak angular velocity and timing of the peak angular velocity of the lumbar spine, and bat tip peak velocity and timing of the peak velocity.

Subsequently, further analyses were performed to compare the lumbar angle throughout the bat swing (Oshikawa et al. 2020). Lumbar angle data were converted into 100 data points for each phase to normalize the differing number of data points for each swing. The 100 data points for each phase were evenly divided into four subphases (I–IV) of 25 data points each. In total, a single bat swing motion consisted of 16 subphases with 400 data points. Finally, the mean angle in each subphase was calculated.

### EMG analysis

The recorded EMG data were analyzed using biomedical information software (BIMUTAS-Video, Kissei Comtec Co., Ltd., Japan). Raw EMG data were band-pass filtered between 20 and 450 Hz to remove motion artifacts. The EMG data in each bat swing phase were presented as the root mean square (RMS) amplitude. The RMS amplitude was normalized as a percentage of the highest RMS amplitude obtained over a 1-second period during the MVIC tests (%MVIC).

### Statistical analysis

The data on all the analysis outcomes except for the STAI Y-1 score were represented as the mean values of the middle three bat swings in each condition, and these mean values were used in the statistical analyses. After confirming all the data with normal distribution by using the Kolmogorov–Smirnov test and homoscedasticity by using the Levene test, one-way ANOVA repeated for each condition was used to compare the STAI Y-1 score, duration of each phase, timing of the five key events during a bat swing, instantaneous peak angle, and ROM of the lumbar spine, peak angular velocity and timing of the peak angular velocity of the lumbar spine, and bat tip peak velocity and timing of the peak velocity. In addition, two-way ANOVA repeated for the conditions and phases, and for the conditions and subphases was used to compare the EMG activity of trunk muscles and lumbar kinematics throughout the bat swing under the three pressure conditions. Bonferroni correction was used as a post hoc test. Partial *η^2^* was calculated for the effect size of one- and two-way ANOVA, with values of ≥0.01, <0.06, ≥0.06, and <0.14, and ≥0.14, indicating small, medium, and large effects, respectively (Cohen 1988). The alpha level was set at 0.05. All statistical analyses were performed using SPSS Statistics 25.0 (IBM Japan Corp, Japan).

## Results

The significant main effects of the pressure conditions were shown in the STAI Y-1 score (F_(2,26)_ = 36.545, P < 0.001, partial *η^2^ *= 0.738), bat tip peak velocity (F_(2,26)_ = 7.548, P = 0.003, partial *η^2^ *= 0.367), swing phase duration (F_(2,26)_ = 20.257, P < 0.001, partial *η^2^ *= 0.609), timing of the lead foot contacting the ground (F_(2,26)_ = 20.257, P < 0.001, partial *η^2^ *= 0.609), instantaneous peak lumbar flexion angle (F_(2,26)_ = 18.423, P < 0.001, partial *η^2^ *= 0.606), ROM of lumbar extension/flexion (F_(2,26)_ = 26.935, P < 0.001, partial *η^2^ *= 0.692), peak angular velocity of lumbar extension (F_(2,26)_ = 9.831, P = 0.003, partial *η^2^ *= 0.450), flexion (F_(2,26)_ = 6.795, P = 0.019, partial *η^2^ *= 0.362), and lateral flexion toward the back side (F_(2,26)_ = 9.771, P = 0.001, partial *η^2^ *= 0.449).

The results of the post hoc tests are as follows: The STAI Y-1 score was significantly higher under the pressure and emphasized pressure conditions than under the non-pressure condition (P < 0.001; Table 2). The bat tip peak velocity was significantly higher in the emphasized pressure condition than in the non-pressure condition (P = 0.005, Table 3). The pressure and emphasized pressure conditions yielded significantly shorter swing phase duration and time between the lead foot contacting the ground and the assumed ball contact than did the non-pressure condition (P < 0.003 and 0.003, Table 4). The instantaneous peak lumbar flexion angle and ROM of lumbar extension/flexion were significantly larger under the pressure and emphasized pressure conditions than under the non-pressure condition (P < 0.002 and 0.001, respectively; Table 5). The peak angular velocity of lumbar extension, and flexion and lateral flexion toward the back side were significantly higher under the pressure and emphasized pressure conditions than under the non-pressure condition (P < 0.020, 0.049, and 0.006, respectively; Table 6).

**Table 2.** STAI Y-1

	Non-pressure	Pressure	Emphasized pressure	F_(2,26)_	P value	Effect size (partial *η^2^*)
STAI Y-1 (score)	32.4 ± 6.4	45.4 ± 6.9^a^	44.4 ± 7.1^a^	36.545	<0.001*	0.738

^a^Significantly higher than that under the non-pressure condition.

*P < 0.05, comparison by one-way analysis of variance.

**Table 3.** Bat tip peak velocity and timing of the bat tip peak velocity

	Non-pressure	Pressure	Emphasized pressure	F_(2,26)_	P value	Effect size (partial *η^2^*)
Bat tip peak velocity (m/s)	30.4 ± 3.2	32.6 ± 4.7	34.1 ± 3.1^a^	7.548	0.003*	0.367
Timing of the bat tip peak velocity (ms)^b^	4.4 ± 21.7	2.3 ± 24.5	2.9 ± 24.0	0.293	0.748	0.022

^a^Significantly higher than that under the non-pressure condition.

^b^Zero ms indicates the assumed ball contact in the timing of the bat tip peak velocity.

*P < 0.05, comparison by one-way analysis of variance.

**Table 4.** Duration of each phase and timing of the five key events during the bat swing

	Non-pressure	Pressure	Emphasized pressure	F_(2,26)_	P value	Effect size (partial *η^2^*)
Duration of phase						
Preparation (ms)	592.7 ± 180.2	625.2 ± 172.0	658.8 ± 164.2	2.181	0.133	0.144
Weight shift (ms)	516.7 ± 144.6	550.7 ± 175.0	525.5 ± 169.6	1.196	0.319	0.084
Swing (ms)	257.9 ± 48.7	219.0 ± 33.5^b^	212.5 ± 28.4^b^	20.257	<0.001*	0.609
Follow-through (ms)	219.9 ± 80.8	215.1 ± 68.4	209.8 ± 62.3	0.508	0.506	0.038
Timing of the five key events^a^						
Start (ms)	−1367.3 ± 275.7	−1395.0 ± 296.2	−1396.8 ± 260.5	0.448	0.644	0.033
Highest point of the lead toe (ms)	−774.5 ± 165.0	−769.8 ± 186.5	−738.0 ± 180.2	1.376	0.270	0.096
Lead foot contact with the ground (ms)	−257.9 ± 48.7	−219.0 ± 33.5 ^c^	−212.5 ± 28.4 ^c^	20.257	<0.001*	0.609
Assumed ball contact (ms)	0	0	0	-	-	-
Finish (ms)	219.9 ± 80.8	215.1 ± 68.4	209.8 ± 62.3	0.508	0.506	0.038

^a^Zero ms indicates the assumed ball contact in the timing of the peak angular velocity.

^b^Significantly shorter than that under the non-pressure condition.

^c^Significantly closer to the assumed ball contact timing than that under the non-pressure condition.

*P < 0.05, comparison by one-way analysis of variance.

**Table 5.** Instantaneous peak angle and range of motion (ROM) of the lumbar spine

	Non-pressure	Pressure	Emphasized pressure	F_(2,26)_	P value	Effect size (partial *η^2^*)
Lumbar extension (°)	−3.9 ± 10.4	−2.6 ± 13.6	−4.1 ± 13.4	0.227	0.799	0.019
Lumbar flexion (°)	27.9 ± 8.0	32.4 ± 9.4^a^	35.3 ± 9.8^a^	18.423	<0.001*	0.606
ROM of lumbar extension/flexion (°)	24.1 ± 7.1	29.8 ± 8.1^a^	31.2 ± 8.8^a^	26.935	<0.001*	0.692
Lumbar rotation toward the lead side (°)	11.6 ± 3.9	12.1 ± 3.7	15.1 ± 6.5	3.369	0.076	0.219
Lumbar rotation toward the back side (°)	8.8 ± 6.3	10.2 ± 5.7	8.5 ± 5.6	1.573	0.228	0.116
ROM of lumbar rotation (°)	20.4 ± 5.4	22.3 ± 5.1	23.6 ± 8.5	3.119	0.062	0.206
Lumbar lateral flexion toward the lead side (°)	8.9 ± 3.7	8.1 ± 3.6	8.8 ± 4.0	0.718	0.461	0.056
Lumbar lateral flexion toward the back side (°)	16.9 ± 4.2	18.8 ± 4.4	17.8 ± 6.3	0.874	0.430	0.068
ROM of lumbar lateral flexion (°)	25.8 ± 5.1	26.9 ± 5.1	26.5 ± 6.7	0.298	0.745	0.024

^a^Significantly larger than that under the non-pressure condition.

*P < 0.05, comparison by one-way analysis of variance

**Table 6.** Peak angular velocity and timing of peak angular velocity of the lumbar spine

	Non-pressure	Pressure	Emphasized pressure	F_(2,26)_	P value	Effect size (partial *η^2^*)
Peak angular velocity						
Lumbar extension (°/s)	234.4 ± 69.4	278.0 ± 72.4^a^	285.4 ± 84.7^a^	9.831	0.003*	0.450
Lumbar flexion (°/s)	170.9 ± 56.4	208.3 ± 63.0^a^	235.1 ± 97.1^a^	6.795	0.019*	0.362
Lumbar rotation toward the lead side (°/s)	348.7 ± 99.0	360.2 ± 84.4	393.0 ± 116.2	1.796	0.201	0.130
Lumbar rotation toward the back side (°/s)	226.0 ± 108.8	276.0 ± 98.1	312.3 ± 154.0	4.369	0.072	0.267
Lumbar lateral flexion toward the lead side (°/s)	218.5 ± 84.0	236.9 ± 75.4	246.4 ± 100.1	1.897	0.172	0.136
Lumbar lateral flexion toward the back side (°/s)	206.2 ± 62.1	250.4 ± 51.1^a^	253.9 ± 73.4^a^	9.771	0.001*	0.449
Timing of peak angular velocity^b^						
Lumbar extension (ms)	22.7 ± 46.7	34.4 ± 40.3	30.3 ± 41.3	0.965	0.395	0.074
Lumbar flexion (ms)	−186.4 ± 24.8	−181.0 ± 19.7	−181.9 ± 23.2	0.594	0.560	0.047
Lumbar rotation toward the lead side (ms)	67.7 ± 13.5	59.7 ± 28.2	71.6 ± 17.8	1.044	0.368	0.080
Lumbar rotation toward the back side (ms)	−69.6 ± 64.1	−54.5 ± 44.3	−55.6 ± 41.8	0.682	0.515	0.054
Lumbar lateral flexion toward the lead side (ms)	112.0 ± 28.6	92.9 ± 73.2	107.3 ± 42.2	0.786	0.417	0.061
Lumbar lateral flexion toward the back side (ms)	−131.8 ± 38.4	−106.9 ± 65.5	−128.9 ± 34.3	1.164	0.310	0.088

^a^Significantly higher than that under the non-pressure condition.

^b^Zero ms indicates the assumed ball contact in the timing of the peak angular velocity.

*P < 0.05, comparison by one-way analysis of variance.

**Figure 3.**
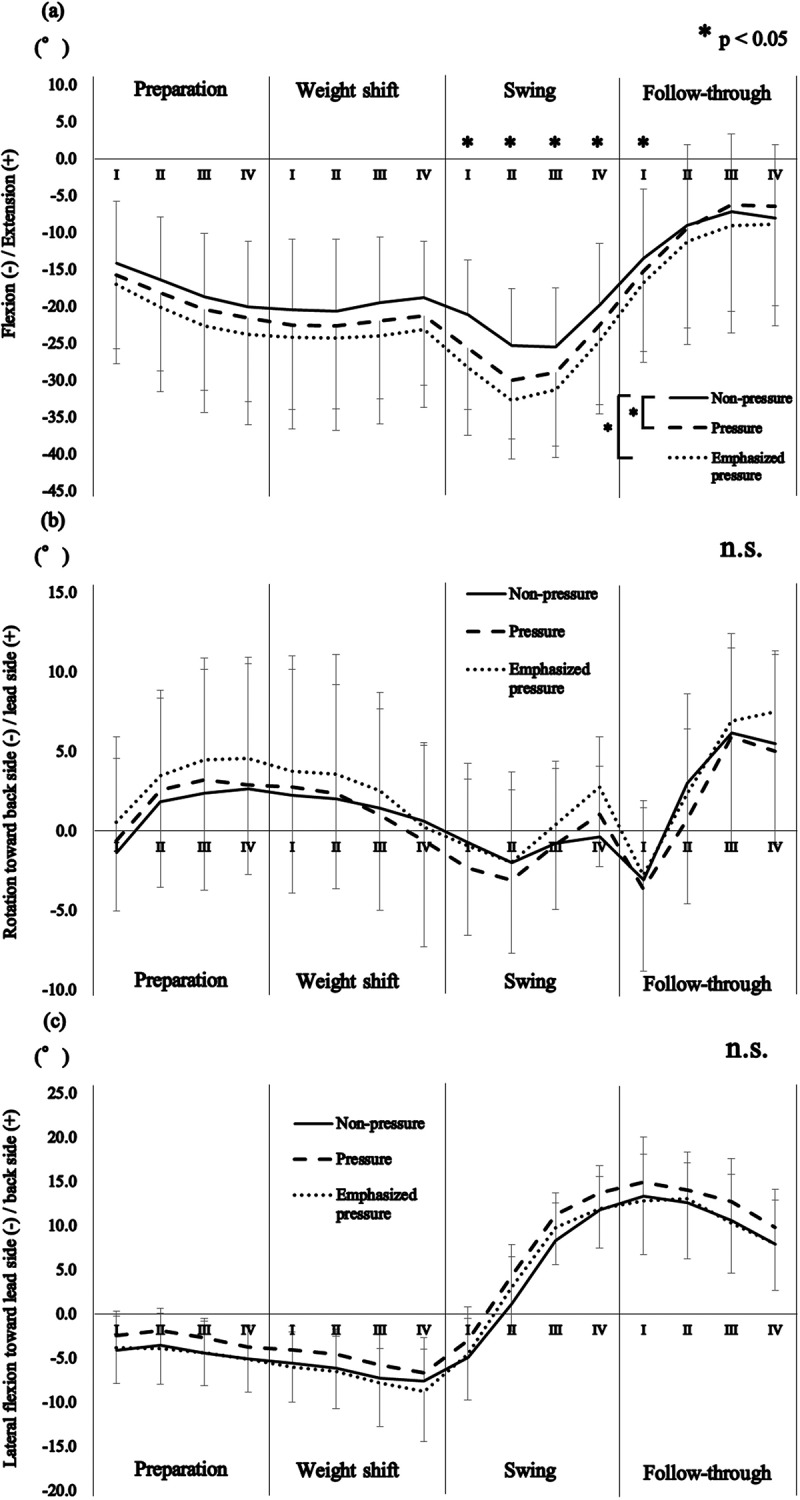
Mean and standard deviation of the lumbar kinematics throughout the bat swing relative to (a) extension/flexion, (b) rotation, and (c) lateral flexion. Zero degrees indicates the mean lumbar angles relative to the pelvis during the anatomical standing posture. *P < 0.05. n.s., no significant difference

A significant interaction was found in the lumbar extension/flexion angle throughout the bat swing between the conditions and the subphases (F_(30,390)_ = 3.904, P = 0.002, partial *η^2^ *= 0.245). The post hoc tests revealed that the pressure and emphasized pressure conditions resulted in significantly greater lumbar flexion than the non-pressure condition, from swing phase I to the follow-through phase I (P < 0.029; Figure 3(a)).

**Figure 4.**
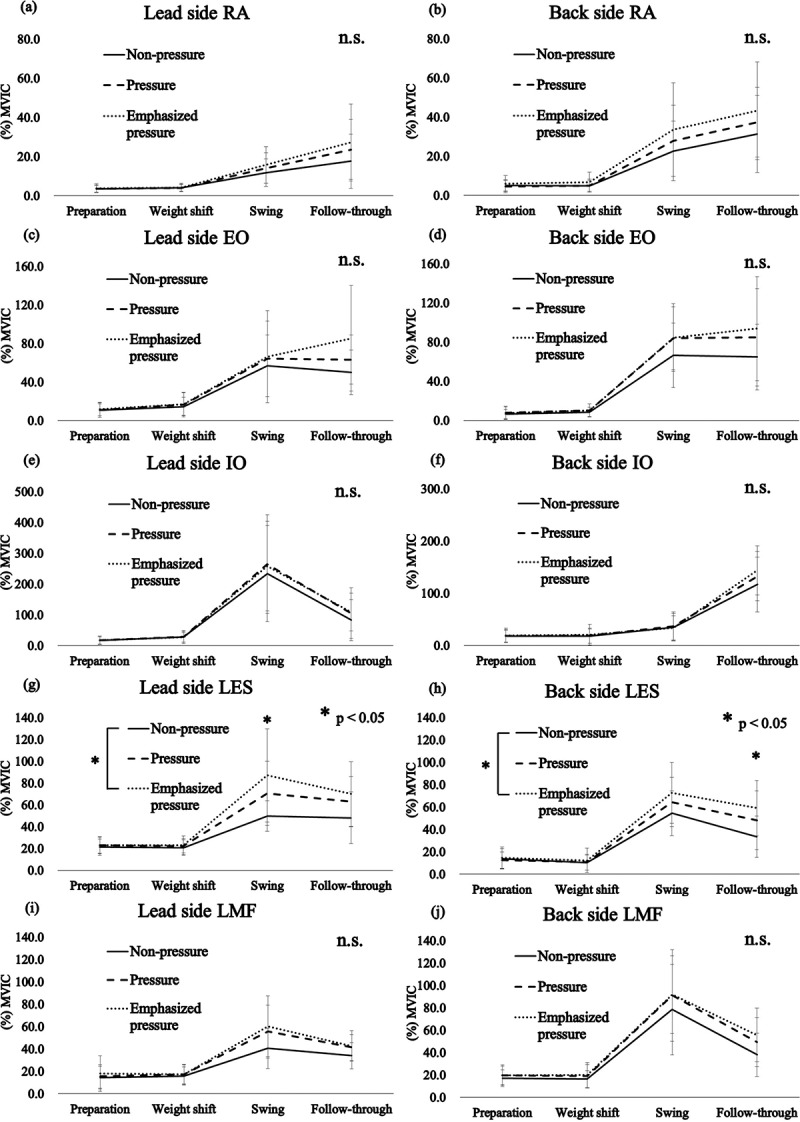
Mean and standard deviation for the electromyographic activity of the trunk muscles with regard to (a) and (b) the rectus abdominis (RA), (c) and (d) external oblique (EO), (e) and (f) internal oblique (IO), (g) and (h) lumbar erector spinae (LES), and (i) and (j) lumbar multifidus (LMF) muscles. *P < 0.05. n.s., no significant difference; MVIC, maximal voluntary isometric contraction

In the EMG activity of the trunk muscles, significant interactions were found on both sides of the LES between the conditions and the phases (lead side: F_(6,78)_ = 2.015, P = 0.036, partial *η^2^ *= 0.094, back side: F_(6,78)_ = 2.788, P = 0.026, partial *η^2^ *= 0.125). The post hoc test results demonstrated that the LES activities of the lead and back sides in the swing and follow-through phases were significantly higher under the emphasized pressure condition than under the non-pressure condition (P = 0.009 and 0.019, respectively; Figure 4(g,h)).

## Discussion

This study aimed to examine the influence of psychological pressure on the lumbar kinematics and EMG activity of the trunk muscles during the batting action. The STAI Y-1 scores indicate that the participants were affected by the different psychological pressure conditions on bat tip velocity.

The instantaneous peak lumbar flexion angle, lumbar ROM in the sagittal plane, and lumbar flexion angle from swing phase I to the follow-through phase I were larger under the pressure and emphasized pressure conditions than under the non-pressure condition. The lumbar spine with physiological lordosis has been reported to be linearized by lumbar flexion toward ball impact during a bat swing (Oshikawa et al. 2020). As this lumbar linearization enables efficient rotation of the pelvis, lumbar spine, and thorax (Welch et al. 1995; Escamilla et al. 2009), it may contribute to increased bat tip velocity and shorter swing phase duration. However, rapid lumbar rotation in the large lumbar flexion position could increase the shearing stress on the lumbar intervertebral disc (Schmidt et al. 2009). Considering the high prevalence of lumbar disc degeneration among baseball players (60%) (Hangai et al. 2009), repeated bat swings with full effort might advance the degeneration of lumbar intervertebral discs.

The peak angular velocity of lumbar extension, and flexion and lateral flexion toward the back side were higher under the pressure and emphasized pressure conditions than under the non-pressure condition. In addition, the LES activities of the lead and back sides in the swing and follow-through phases were higher under the emphasized pressure condition than under the non-pressure condition. In the swing phase, the lumbar rotation toward the lead side was accelerated, and the peak angular velocity of the lumbar flexion and lateral flexion toward the back side were observed. Therefore, we suggest that the high LES activity on the lead side in the swing phase under the emphasized pressure condition contributed to the acceleration of lumbar rotation toward the lead side, and the control of the rapid lumbar flexion and lateral flexion toward the back side by its eccentric contraction. Conversely, in the follow-through phase, the lumbar rotation toward the lead side was decelerated, and the peak angular velocity of lumbar extension was observed. Thus, the high LES activity on the back side in the follow-through phase under the emphasized pressure condition may have contributed to the deceleration of the lumbar rotation toward the lead side by its eccentric contraction and rapid lumbar extension. From the result of the LES activity, repeated bat swings with the player’s full effort increase loads to the LES and might cause a muscular LBP.

Contrary to our hypothesis, there was no change in the instantaneous peak angle of the lumbar rotation, the peak angular velocity of the lumbar rotation toward both the lead and back sides, and the EO and IO activities under the psychological pressure conditions. It is feasible to suggest that the instantaneous peak angle of the lumbar rotation and peak angular velocity of the lumbar rotation toward both the lead and back sides did not increase because the EO and IO activities associated with lumbar rotation were already extremely high in the non-pressure condition, and could not increase in the pressure conditions.

There were three limitations in this study. First, we did not measure heart rate, which is an index for objective evaluation of psychological pressure. Second, the trials of each condition could not be performed randomly because the psychological pressure must be increased gradually. Therefore, the outcomes of the pressure and emphasized pressure conditions might be affected by the bat swings before each condition. Finally, the participants in this study performed only five practice swings under each condition. Baseball players perform baseball batting many times during practice; thus, these conditions must be examined in future studies.

In conclusion, the lumbar flexion angle, peak angular velocities of lumbar flexion and lateral flexion toward the back side, and LES activities on the lead side in the swing phase and back side on the follow-through phase increased under the psychological pressure conditions. These results indicate that the baseball batting action under psychological pressure influenced the lumbar kinematics and bilateral LES activities, and may be related to the development of LBP.
